# Exosome-like Nanoparticles Extracted from Plant Cells for Diabetes Therapy

**DOI:** 10.3390/ijms26189155

**Published:** 2025-09-19

**Authors:** Xin Xiao, Yuliang Guo, Nontokozo Zimbili Msomi, Md. Shahidul Islam, Maoquan Chu

**Affiliations:** 1 School of Life Sciences and Technology, Tongji University, Shanghai 200092, China; xinxiao_josh@tongji.edu.cn (X.X.);; 2 Department of Biochemistry, School of Life Sciences, University of KwaZulu-Natal, Pietermaritzburg 3209, South Africa; 3 Department of Biochemistry, University of KwaZulu-Natal, Westville Campus, Durban 4000, South Africa

**Keywords:** plant-derived exosome-like nanoparticles, diabetes and its complications, natural nanocarriers, nanobiomedicine, precision therapy

## Abstract

Diabetes mellitus (DM) is a complex metabolic disorder characterized by chronic hyperglycemia and associated complications such as cardiovascular disease, nephropathy, retinopathy, neuropathy, and chronic non-healing wounds. Current antidiabetic therapies offer only partial glycemic control and are limited by poor bioavailability, adverse effects, and an inability to prevent disease progression. Plant-derived exosome-like nanoparticles (PENPs) have emerged as a promising class of natural nanocarriers with excellent biocompatibility, low immunogenicity, and intrinsic multi-component bioactivity. However, few reviews have addressed recent progress in PENPs for DM therapy. To capture the recent developments in this area, this review provides a systematic synthesis of recent advances in PENPs for DM therapy, covering plant sources, extraction and purification methods, molecular compositions, and therapeutic mechanisms. Preclinical studies have demonstrated that PENPs can improve hyperglycemia, enhance insulin sensitivity, regulate hepatic lipid metabolism, and promote wound healing by modulating oxidative stress, inflammation, gut microbiota, glucose metabolism, and insulin signaling. Additionally, PENPs have been shown to promote angiogenesis via glycolytic reprogramming. Despite these promising findings, challenges including scalable isolation, standardized physicochemical characterization, and clinical translation remain. Future directions include engineering multifunctional PENPs, establishing Good Manufacturing Practice (GMP)-compliant production, and conducting clinical trials to facilitate their integration into precision therapeutics for diabetes management.

## 1. Introduction

Diabetes mellitus (DM) has emerged as a critical global health burden [1]. According to the International Diabetes Federation (IDF, 2025) Atlas, approximately 589 million adults aged 20–79 worldwide are affected by diabetes, with this figure projected to increase to 853 million by 2050 [2]. The vast majority of these cases are classified as type 2 diabetes mellitus (T2DM). Chronic hyperglycemia associated with T2DM can lead to a range of debilitating complications, including cardiovascular disease, chronic kidney disease, retinopathy, neuropathy, and impaired wound healing [3]. These complications are fundamentally linked to pathological mechanisms such as insulin resistance, pancreatic β-cell dysfunction, and the dysregulation of the oxidative stress–inflammation axis [4,5]. Despite considerable advances in therapeutic approaches, current interventions remain limited in their ability to achieve sustained glycemic control, prevent long-term complications, and reverse disease progression [6,7].

Standard treatments for T2DM include insulin and metformin, alongside newer agents such as glucagon-like peptide-1 (GLP-1) receptor agonists and sodium–glucose cotransporter 2 inhibitors [8]. While these agents effectively reduce blood glucose levels, they are hindered by limitations including adverse side effects, low patient compliance, and the necessity for lifelong administration, despite offering ancillary benefits such as cardiovascular protection and weight loss [9,10]. For instance, metformin has limited oral bioavailability and is commonly associated with gastrointestinal disturbances [11,12], whereas insulin requires frequent injections, reducing patient adherence [13]. Although nanotechnology-based therapeutics have shown promise in enhancing delivery efficiency and targeting specificity, many synthetic nanomaterials face significant challenges in clinical translation due to issues such as immunogenicity, potential toxicity, and scalability barriers [14,15,16]. This has prompted a shift toward the development of safer, more biocompatible, and sustainable bio-nanocarriers.

Exosomes are nano-sized extracellular vesicles first described by Johnstone et al. in 1987 [17], and have since been identified in various mammalian cell types and biological fluids, including stem cells, tumor cells, immune cells, blood, and urine [18]. Mammalian-derived exosomes (MDEs) are recognized for their role in intercellular communication, immune modulation, and pathological progression and as carriers for therapeutic molecules [19]. However, their clinical utility is hampered by technical constraints such as low yield, complex isolation procedures, and potential immunological risks stemming from their heterogeneous cargo [20]. Consequently, increasing attention has been directed toward PENPs, which offer a relatively accessible and potentially scalable, immunologically safer alternative compared to mammalian exosomes [21].

The field of plant-derived exosomes was initiated in 2009, when Regente et al. reported the presence of exosome-like vesicles in sunflower seedlings using transmission electron microscopy and proteomic analysis [22]. PENPs closely resemble MDEs in terms of size, surface charge, density, and certain molecular components [23]. More importantly, PENPs offer several intrinsic advantages, such as high yield, low immunogenicity, simple extraction procedures, and environmental sustainability [24]. Furthermore, PENPs have been derived from a variety of edible and medicinal plant sources [25,26] and exhibit pharmacological activities comparable to mammalian-derived exosomes, including antifibrotic [27], antiviral [28], and anticancer [29,30,31,32] effects. Notably, PENPs are enriched with bioactive microRNAs (miRNAs), which can be efficiently internalized by mammalian cells and are implicated in regulating host cellular metabolism and signaling pathways [33,34,35].

Emerging evidence suggests that PENPs modulate oxidative stress [36], inflammatory responses [37,38], gut microbiota homeostasis [39], glucose metabolism [40], and insulin signaling [41], collectively contributing to improved glycemic control and enhanced tissue repair in diabetic models. These findings highlight the potential of PENPs as a novel, multi-target therapeutic platform for diabetes management. Nevertheless, their clinical readiness remains limited due to the absence of standardized isolation and characterization protocols, difficulties in achieving industrial-scale and GMP-compliant manufacturing, insufficient mechanistic understanding, and unresolved regulatory frameworks [42].

**Figure 1:**
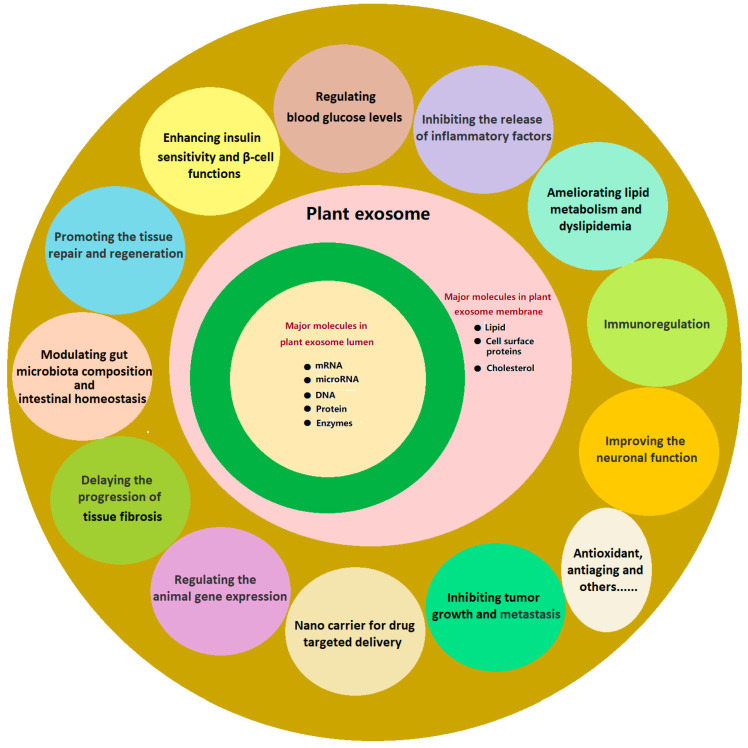
Therapeutic roles and bioactive components of PENPs. PENPs are natural nanocarriers composed of a lipid bilayer containing lipids, proteins, and cholesterol and enriched with internal cargos such as mRNA, microRNA, DNA, proteins, and enzymes. They exert multiple therapeutic effects by enhancing insulin sensitivity, regulating glucose and lipid metabolism, modulating gut microbiota and intestinal homeostasis, promoting tissue repair, and reducing inflammation and oxidative stress. PENPs also contribute to neuroprotection and antifibrotic and antitumor activities and support targeted delivery of therapeutic agents.

This review aims to provide a comprehensive overview of recent advancements in the field, focusing on (i) the biogenesis, structural characteristics, and in vivo behavior of PENPs; (ii) their molecular mechanisms in regulating metabolic and inflammatory pathways; (iii) preclinical evidence supporting their therapeutic efficacy and delivery advantages; and (iv) existing bottlenecks and prospective strategies for clinical translation. Unlike previous reviews that broadly surveyed PENPs in diverse biomedical contexts [24,42], the present work concentrates specifically on diabetes and its complications, thereby offering a focused perspective. It integrates mechanistic insights with therapeutic evidence to illustrate how PENPs modulate diverse processes relevant to diabetes, such as glucose and lipid metabolism, oxidative stress, inflammation, gut microbiota, and tissue repair. By synthesizing current knowledge, this review seeks to establish a scientific foundation for the future application of PENPs in precision diabetes therapy. Figure 1 provides a conceptual overview of their multifunctional roles and bioactive components, highlighting their therapeutic potential in diabetes and related complications.

## 2. Biogenesis Mechanisms of PENPs

According to the Food and Agriculture Organization of the United Nations, more than 50,000 plant species with pharmacological potential have been identified worldwide, offering a vast natural reservoir for the development of PENPs [43]. To date, PENPs have been successfully isolated from a broad spectrum of sources, including fruits (grape [44], lemon [45], apple [46] and grapefruit [47]), vegetables (ginger [48], broccoli [49], and *Momordica charantia* (bitter melon) [50]), and medicinal herbs (*Panax ginseng* [51], *Pueraria lobata* [52], *Lonicera japonica* [53], *Portulaca oleracea* [54]). This widespread occurrence suggests that the formation and secretion of PENPs may represent a conserved and biologically significant process across diverse plant taxa. Although the precise mechanisms underlying PENP biogenesis have not yet been fully elucidated, current evidence indicates the involvement of three primary pathways: (i) the classical endosomal route involving multivesicular bodies (MVBs), (ii) a vacuole-associated pathway typically activated under biotic or abiotic stress conditions, and (iii) an unconventional secretory pathway mediated by exocyst-positive organelles (EXPOs) [55]. The relative contribution of these pathways may differ across plant species and developmental stages, underscoring the necessity for further systematic investigation. Among these, the MVB-dependent pathway is widely considered the predominant route for PENP formation and shares considerable structural and regulatory similarity with mammalian exosome biogenesis.

**Figure 2:**
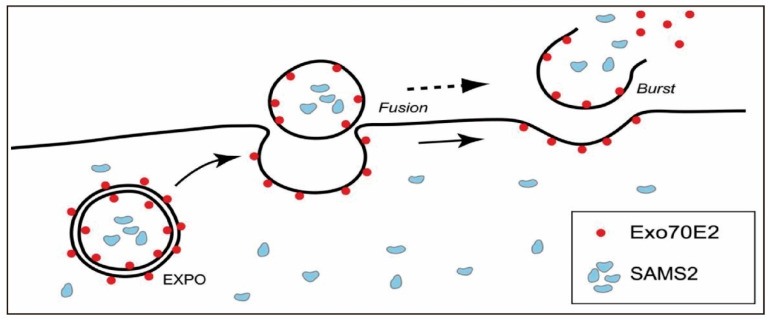
Schematic model of EXPO-mediated unconventional secretion. EXPOs fuse with the plasma membrane and release single-membrane vesicles into the apoplast, where they rupture and deliver cytosolic proteins such as SAMS2 to the cell wall region [58]. Solid arrows indicate the progression of EXPO vesicles from formation in the cytoplasm to transport toward the plasma membrane, while the dashed arrow denotes the membrane fusion and burst step enabling extracellular release.

As early as the 1960s, MVBs were observed in carrot cell cultures fusing with the plasma membrane to release vesicles, providing early evidence of their role in extracellular vesicle secretion [56]. Subsequent studies have confirmed this phenomenon in various plant species, thereby reinforcing the central role of the MVB pathway in PENP production. In the MVB-mediated process, early endosomes are first generated via plasma membrane invagination. The resulting endosomes subsequently incorporate molecular cargos from intracellular organelles such as the endoplasmic reticulum and trans-Golgi network. These cargos are then sorted into intraluminal vesicles, culminating in the formation of mature MVBs. In plant cells, this process is primarily governed by the endosomal sorting complex required for transport machinery, which orchestrates key steps such as ubiquitin recognition, vesicle budding, and membrane scission. Mature MVBs may either undergo lysosomal degradation or, alternatively, be directed by Rab GTPases and cytoskeletal elements, namely microtubules and actin filaments to fuse with the plasma membrane and release PENPs into the extracellular milieu. Once secreted, these PENPs mediate crucial intercellular and potentially interspecies communication. Beyond the canonical MVB route, a vacuole-mediated secretion mechanism may be activated under stress conditions, particularly during pathogen attack. In such scenarios, vacuoles enriched with hydrolytic enzymes and defense-related proteins may fuse with the plasma membrane, releasing their contents into the apoplast to enhance host defense. In grapefruit (*Citrus paradisi*) epidermal cells, localization analyses have confirmed the presence of PENPs within central vacuoles [57]. Moreover, ultrastructural observations have revealed membrane contact sites between MVBs and small vacuoles, suggesting potential crosstalk or convergence between these two pathways. Taken together, these findings indicate that the vacuole-associated pathway is mainly engaged under stress conditions while the MVB-mediated route appears to be the principal mechanism governing constitutive secretion in PENPs derived from fruits and leaves. The EXPO-mediated pathway represents a non-canonical, double-membrane-based secretory mechanism that enables the direct export of cytoplasmic contents to the cell wall region. This process is illustrated in Figure 2 [58], which shows the membrane fusion and extracellular release steps of EXPO-mediated secretion. Unlike autophagosomes or endosomes, EXPOs are structurally distinct and do not require autophagic or starvation signals for activation. However, the physiological relevance of EXPOs in PENP biogenesis remains largely unexplored, and current research in this area is still in its infancy. Most available evidence comes from studies in model species such as *Arabidopsis thaliana* [58] and remains largely descriptive with little functional validation. Thus, the extent to which EXPOs constitute a conserved pathway versus a lineage-specific adaptation remains to be determined. Clarifying this uncertainty will require rigorous comparative and functional studies, thereby providing critical insight into their role in PENP biogenesis.

Overall, existing studies support the view that the MVB pathway serves as the predominant mechanism for constitutive PENP secretion across diverse plant species whereas the vacuole-associated route functions mainly as a stress-inducible and context-dependent alternative. By contrast, the EXPO pathway remains insufficiently characterized, and its evolutionary conservation has yet to be established.

## 3. Structural and Compositional Characteristics of PENPs

### 3.1. Proteins

PENPs are enriched with a diverse array of functional proteins, including stress-responsive proteins, defense-associated proteins, annexins, aquaporins, heat shock proteins, metabolic enzymes, and signal transduction mediators [24,59]. These proteins not only contribute to the structural integrity and stability of the vesicle membrane but also participate in intercellular communication and may exert anti-inflammatory and antioxidant effects. Classical surface markers of mammalian extracellular vesicles include CD9, CD63, CD81, and TSG101, widely employed for the identification of mammalian exosomes [22,60]. However, the applicability of these markers to PENPs remains ambiguous and requires further empirical validation.

The proteomic profiles of PENPs vary significantly across plant species, influencing their biological interactions and uptake mechanisms. For instance, lectin-like proteins present on the surface of garlic-derived PENPs have been shown to bind specifically to CD98 receptors on HepG2 cells, thereby facilitating endocytic internalization. Consequently, blocking CD98 markedly reduces uptake efficiency [61]. Similarly, bitter melon-derived PENPs are enriched in antioxidant-related proteins, as indicated by Gene Ontology (GO) and Kyoto Encyclopedia of Genes and Genomes (KEGG) pathway analyses, suggesting that these protein components may contribute directly to their therapeutic efficacy [62]. In addition to species-dependent differences, vesicles obtained from plant suspension cultures also display source-specific protein signatures. For instance, *Stevia rebaudiana* suspension-culture-derived vesicles were enriched in extracellular-matrix-related proteins whereas *Vaccaria hispanica* suspension-culture-derived vesicles predominantly contained cytoplasmic proteins, highlighting the influence of culture conditions on PENP protein composition [63]. Nevertheless, proteomic studies on PENPs remain at an early stage. Comprehensive protein characterization and mechanistic exploration across various plant sources are critical to advancing the precision application of PENPs in disease modulation and targeted therapy [42].

### 3.2. Lipids

Lipids form the structural backbone of PENPs and play essential roles in vesicle biogenesis, membrane stability, transmembrane transport, and cellular recognition. Recent studies have identified that PENPs are typically rich in phospholipids such as phosphatidic acid (PA), phosphatidylethanolamine (PE), phosphatidylcholine (PC), and plant-specific glycolipids including digalactosyldiacylglycerol (DGDG) and monogalactosyldiacylglycerol (MGDG). The composition and abundance of lipid species vary markedly among plant-derived sources, resulting in differences in biodistribution, cellular uptake, and functional properties. For example, grapefruit-derived PENPs are predominantly composed of PE and PC (approximately 46% and 29%, respectively) and exhibit enhanced intestinal absorption with preferential hepatic accumulation [64]. Ginger-derived PENPs contain abundant PA (~38%), together with DGDG (~33%) and MGDG (~21%), and show prominent uptake by small intestinal epithelial cells following oral administration [65]. Oat-derived PENPs are enriched in PC (~30%) and DGDG (~29.8%) and have been shown to cross the blood–brain barrier (BBB) and to be preferentially taken up by microglial cells, thereby exerting neuroprotective effects [66]. Notably, ginseng-derived PENPs are enriched in digalactosylmonoacylglycerol (DGMG, ~59.4% of total lipids); however, whether this unique glycolipid composition contributes to their gastrointestinal distribution remains to be clarified [32]. To enable direct comparison, Table 1 summarizes lipid profiles and associated in vivo targeting across representative plant sources.

Lipid composition also modulates interactions with the gut microbiota. Experimental evidence indicates that PA-enriched PENPs are preferentially internalized by *Lactobacillus* species, with ginger-derived PENPs as a typical example, whereas PC-dominant PENPs are more readily taken up by *Ruminococcus* [39,67]. This suggests that specific lipid moieties may serve as molecular “recognition signals” for microbiota communication and targeting.

In addition to structural and targeting functions, lipid constituents of PENPs have demonstrated intrinsic therapeutic activities. For instance, PA-enriched ginger-derived PENPs exhibit antimicrobial effects by binding to the hemin-binding protein 35 (HBP35) of *Porphyromonas gingivalis*, which in turn inhibits the pathogenicity of this periodontal bacterium [68]. In addition, they upregulate the expression of Foxa2 in intestinal epithelial cells, thereby ameliorating insulin resistance induced by a high-fat diet [65]. DGDG-enriched oat-derived PENPs have been shown to disrupt Dectin-1 receptor binding to β-glucan, modulating immune signaling and attenuating neuroinflammation in an alcohol-induced brain injury model [66].

Notably, lipid composition can be influenced by extraction conditions, including buffer pH and endogenous phospholipase activity. For example, ginger-derived PENPs purified at acidic pH (4–5) exhibited more intense lipid-band signals and higher polyphenol content, suggesting a pH-dependent enrichment of certain lipid classes and improved vesicle yield [69]. Furthermore, lipids extracted from PENPs have been successfully reconstituted into synthetic vesicles. Grapefruit-derived lipid nanocarriers loaded with a STAT3 inhibitor have demonstrated efficient targeting to glioma cells [70], highlighting the potential of PENP-derived lipids as bioactive components in precision nanomedicine platforms.

### 3.3. Nucleic Acids

PENPs are enriched with small RNAs, particularly miRNAs, which function as key non-coding regulators in intercellular signaling and gene expression modulation [71]. miRNAs exert their effects by base-pairing with complementary sequences in target mRNAs, leading to transcript degradation or translational repression across various biological pathways. Notably, the miRNA profiles of PENPs differ substantially from those of their parental plant tissues, suggesting the presence of selective miRNA sorting mechanisms. For example, ginger-derived PENPs exhibit a distinct miRNA signature compared to native ginger tissues, indicating that miRNA incorporation is not random [72]. Emerging studies have proposed that specific sequence motifs or autophagy-related proteins such as LC3 may participate in the selective loading of miRNAs into PENPs [73,74].

Remarkably, plant-derived miRNAs can mediate cross-kingdom regulation when delivered via PENPs. Several plant miRNAs have been identified in human serum, where they modulate mammalian gene expression in a manner analogous to endogenous miRNAs [33]. For instance, certain plant miRNAs target genes such as *LDLRAP1*, thereby influencing lipid metabolism. Chapado et al. predicted that miRNAs such as *miR156a*, *miR167a*/*b*/*c*, and *miR166a*/*b*/*c*/*d*/*e* from broccoli could regulate human genes associated with immunity and metabolism, including *PIK3R1* and *STAT1*, potentially impacting metabolic inflammation [34]. Blueberry-derived PENPs contain miRNAs such as *miR-162* and *miR-156e*, which are predicted to target signaling molecules like MAPK and PDE7A, thus participating in chronic inflammation modulation [35].

**Figure 3:**
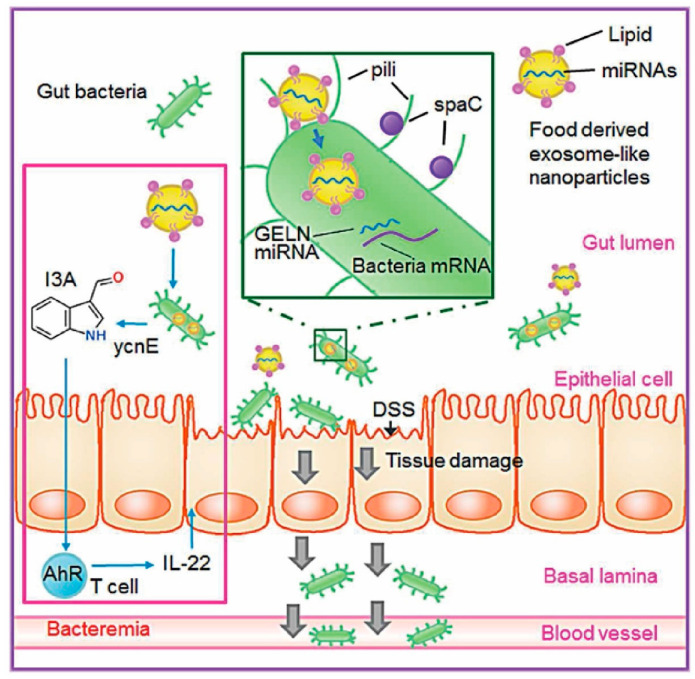
Schematic representation of the cross-kingdom regulatory mechanism by which ginger-derived PENPs deliver miRNAs to gut microbes. In addition to *mdo-miR7267-3p*-mediated targeting of *ycnE* in Lactobacillus, other miRNAs such as *ath-miR167a* inhibit microbial adhesion via *spaC*, collectively enhancing microbial metabolic output, activating AhR signaling, and promoting IL-22 dependent intestinal protection [39].

Additionally, *Osa-miR-530-5p* from ginger PENPs has been shown to bind the ribosomal frameshifting site of the SARS-CoV-2 *ORF1ab* gene, thereby suppressing viral replication [28]. Another example involves *mdomiR7267-3p* from ginger-derived PENPs, which targets the *ycnE* gene in Lactobacillus, promoting the production of indole-3-aldehyde and stimulating the host’s epithelial expression of the anti-inflammatory cytokine IL-22, thus mitigating intestinal inflammation [39] (Figure 3). Moreover, *miR156a*, expressed in spinach, cabbage, and lettuce, has been reported to directly target the human vascular endothelial *JAM-A* gene, reducing monocyte adhesion and potentially alleviating vascular inflammation associated with atherosclerosis [75].

### 3.4. Functional Small Molecules

PENPs are also rich in functional small molecules and secondary metabolites including gingerols, saponins, isothiocyanates, polysaccharides, and polyphenols that significantly contribute to their bioactivity. For instance, ginger-derived PENPs contain 6-gingerol and 6-shogaol, compounds known for their potent anti-inflammatory properties [76]. Ginseng-derived PENPs are enriched in ginsenoside Rg3, which has been experimentally shown to inhibit melanoma growth by suppressing angiogenesis, indicating that vesicle-mediated delivery enhances the therapeutic potential of active phytochemicals [32]. Lemon-derived PENPs are rich in citric acid and vitamin C, with elevated concentrations of the latter detected within vesicles, contributing to strong antioxidant effects [77].

The accumulation of small molecules in PENPs is influenced by plant species, tissue type, and extraction parameters, all of which can alter the encapsulated metabolite profiles. For example, different density fractions of ginger-derived PENPs contain varying amounts of shogaol, suggesting a relationship between vesicle density and compound loading specificity. In another study, sulforaphane was selectively enriched in the 100,000× *g* ultracentrifugation fraction of broccoli-derived PENPs, while nearly absent in non-vesicular extracts, highlighting the structural role of PENPs in encapsulating hydrophobic compounds [49].

Encapsulation within PENPs significantly improves the pharmacokinetics and targeting of bioactive compounds. Compared to free molecules, PENP-encapsulated agents exhibit enhanced oral bioavailability, improved gastrointestinal stability, and increased tissue-targeting capability. For instance, in studies, β-glucan, which typically demonstrates poor BBB permeability, was able to penetrate the central nervous system when delivered via oat-derived PENPs, thereby alleviating alcohol-induced neuroinflammation and cognitive impairment [68,78]. Similarly, methotrexate conjugated to grapefruit-derived PENPs retained antitumor efficacy while reducing systemic toxicity, underscoring the promise of PENPs as a biocompatible and efficient platform for drug delivery [64]. Collectively, the diversity, structural functionality, loading specificity, and targeting capability of secondary metabolites encapsulated in PENPs establish a robust foundation for their therapeutic application.

## 4. Extraction and Purification Methods of PENPs

### 4.1. Pre-Treatment of Plant Tissues

The extraction of PENPs requires an initial tissue pre-treatment step to facilitate the release of extracellular vesicles. Two commonly employed strategies are tissue disruption and apoplastic infiltration–centrifugation, each offering distinct advantages in terms of yield and purity [79].

#### 4.1.1. Tissue Disruption Method

This approach involves the mechanical homogenization of plant tissues to produce a crude slurry, followed by differential centrifugation to remove cellular debris and isolate vesicles. It is simple to implement and well-suited for large-scale processing. For example, De Robertis et al. utilized this method in combination with ultrafiltration to extract high concentrations of blueberry-derived PENPs [35]. However, extensive cell rupture may lead to contamination with intracellular components and nonspecific vesicles, thereby compromising the purity and consistency of vesicle composition.

#### 4.1.2. Apoplastic Infiltration–Centrifugation Method

This technique involves the vacuum-assisted infiltration of buffer into the leaf apoplast, followed by low-speed centrifugation to collect extracellular vesicles with minimal intracellular contamination. Rutter et al. successfully employed this method to isolate high-purity PENPs from *Arabidopsis thaliana*, characterized by the presence of putative vesicle marker proteins such as syntaxin Penetration 1 (PEN1) [80]. Due to its high specificity, this method is particularly suitable for mechanistic studies. However, it is limited by relatively low vesicle yield and greater operational complexity and is mainly applicable to certain tissue types such as leaves.

### 4.2. Overview of Extraction and Purification Strategies

A diverse range of strategies has been developed for the extraction and purification of PENPs. Differential centrifugation (DC) remains the most commonly employed initial method, suitable for bulk vesicle recovery [81]. When coupled with density-gradient ultracentrifugation (DGUC), it significantly improves vesicle purity and size homogeneity [82]. Ultrafiltration (UF)—particularly in tangential flow filtration (TFF) configurations—enables the efficient processing of high-viscosity plant extracts and is often integrated with size-exclusion chromatography (SEC) or centrifugation for enhanced separation [83]. Polymer-based precipitation, such as with polyethylene glycol (PEG), offers a convenient and rapid enrichment approach, although the co-precipitation of contaminants may necessitate additional purification steps [84].

Immunoaffinity capture allows for high-specificity isolation based on candidate vesicle surface markers such as plant tetraspanin-8 (TET8), PEN1, and ATP-binding cassette transporter G36 (PEN3) [55,80]; however, its broader application is limited by the availability and cost of suitable plant-derived antibodies [85]. SEC, known for its gentle, non-destructive removal of small molecular contaminants, is well-suited for applications requiring high purity [86]. Asymmetric flow field–flow fractionation (AF4) provides high-resolution and shear-free size fractionation and is increasingly applied in advanced vesicle research [45]. Electrophoresis–dialysis hybrid systems allow for the concurrent elimination of charged impurities while maintaining vesicle structural integrity, making them ideal for small-scale, high-precision applications [87]. Aqueous two-phase systems (ATPSs), based on hydrophilic partitioning, offer gentle, scalable separation but require post-processing to remove residual polymers [88]. Additionally, centrifugation-agarose electrophoresis can effectively reduce soluble protein contamination by retaining vesicles in the gel loading well while allowing smaller impurities to migrate out, which is advantageous for mechanistic studies [89]. Microfluidic platforms and their advanced derivatives including hydrophobic interaction chromatography and immunomagnetic capture techniques enable the rapid, automated processing of small-volume samples. These technologies hold great promise for the high-throughput and industrial-scale production of PENPs. To provide a clearer comparative perspective, the major extraction and purification strategies are summarized in Table 2, highlighting their relative trade-offs in terms of purity, scalability, vesicle integrity, advantages, and limitations.

### 4.3. Extraction Strategy Selection and Yield Enhancement Approaches

Building on these comparative trade-offs (Table 2), the selection of PENP extraction and purification strategies must balance yield, purity, scalability, and application purpose [42]. Differential and density-gradient centrifugation constitute an ideal combination for mechanistic and molecular studies requiring high purity, albeit with increased operational complexity [57,90]. In contrast, ultrafiltration and polymer-based precipitation offer moderate purity but high yields, making them more suitable for large-scale or clinical-grade production [24,91]. SEC and AF4 are particularly advantageous for vesicle size profiling and bioactivity analysis [92,93] while immunoaffinity capture allows for the targeted enrichment of specific vesicle subpopulations [87]. Emerging platforms such as ATPS, electrophoresis–dialysis, and microfluidic technologies provide adaptable solutions for processing high-viscosity or low-volume samples [42,94].

In parallel, yield enhancement strategies must consider both physiological and processing variables. For instance, modulating buffer pH has been shown to promote MVB fusion and enhance vesicle secretion, thereby improving overall extraction efficiency [69]. In addition, biotic stressors such as pathogen exposure have been reported to stimulate extracellular vesicle production in plant systems [80]. Looking forward, the integrated optimization of environmental stimuli and downstream process engineering may enable the efficient, large-scale production of PENPs while preserving their structural integrity and bioactivity [95]. Complementary to downstream optimization, sterile plant-tissue-culture systems, including callus cultures and suspension cells, have emerged as an alternative strategy for generating PENPs. These platforms can serve as standardized sources that improve yield, purity, and reproducibility while minimizing environmental contamination and facilitating quality control [63,96]. This, in turn, could accelerate the clinical translation and therapeutic application of PENPs.

## 5. Physicochemical Characterization of PENPs

The physicochemical characteristics of PENPs are fundamental to their biological activity and therapeutic potential. Key parameters such as particle size, surface charge (zeta potential), and morphology govern in vivo stability, cellular uptake mechanisms, and the transmembrane transport efficiency of PENPs. Reported data indicate that PENPs typically range from 30 to 500 nm in diameter, with zeta potentials varying from neutral to approximately −50 mV [97]. These values are strongly influenced by both the plant source as well as the extraction and purification methodologies employed [98].

Particle size measurement is most commonly performed using dynamic light scattering (DLS) and nanoparticle tracking analysis (NTA). DLS provides rapid estimations of average particle size but suffers from limited resolution in polydisperse samples, where the size difference between particles must exceed a factor of three to be distinguishable [99]. In contrast, NTA offers higher resolution and allows the simultaneous determination of both particle size distribution and concentration within the 10–2000 nm range. However, it is more susceptible to interference from non-vesicular impurities [100].

Morphological characterization is typically conducted using TEM, cryogenic electron microscopy (Cryo-EM), and atomic force microscopy (AFM) [101]. While TEM is widely used for visualizing vesicle morphology, it often reveals cup-shaped or disc-like structures that are now recognized as artifacts of dehydration and fixation processes. Cryo-EM better preserves the native morphology of vesicles under near-physiological conditions. For instance, Ju et al. utilized Cryo-EM to image cabbage-derived PENPs and reported a mean diameter of ~160 nm, with intact spherical morphology [102]. AFM enables the high-resolution three-dimensional surface profiling and quantification of nanomechanical parameters such as membrane elasticity and adhesion forces. It also holds promise for the quantitative analysis of vesicle-associated surface biomarkers [103].

Given that PENP preparations often contain non-vesicular contaminants, sample purity represents a critical determinant of characterization accuracy. For example, You et al. reported that cabbage-derived PENPs reached a particle concentration of approximately 10^10^ particles/mL [44]. In comparison, mammalian extracellular vesicles intended for clinical applications are typically required to achieve concentrations exceeding 10^11^ particles/mL [104] to ensure consistent efficacy and biosafety. This discrepancy underscores the urgent need for standardized purification workflows, comprehensive quality control systems, and application-specific purity thresholds to support the clinical translation of PENPs [105]. In this context, community-driven initiatives such as EV-TRACK and the guidelines of the International Society for Extracellular Vesicles (ISEV) have established valuable frameworks for transparent reporting, methodological standardization, and quality control in extracellular vesicle research. Although originally developed for mammalian EVs, these frameworks are increasingly relevant to PENP research and could serve as a foundation for developing plant-specific standards in the future.

## 6. In Vivo Transport and Biodistribution of PENPs

### 6.1. Cellular Uptake Mechanisms and Intracellular Fate of PENPs

Efficient cellular internalization by mammalian cells is a prerequisite for PENPs to exert biological functions or serve as drug delivery vehicles [23,30]. Multiple studies have demonstrated that food-derived nanostructures, including those from milk and plant extracts, can withstand the harsh gastrointestinal environment and be absorbed by host cells, suggesting that cross-kingdom endocytosis is biologically feasible [64,106]. Building upon this evidence, PENPs have been shown to be internalized by various mammalian cell types, including keratinocytes, T and B lymphocytes, intestinal epithelial cells, and solid tumor cells, indicating broad cross-species uptake capability [47].

Current findings indicate that PENPs enter target cells via three principal pathways. First, PENPs may directly fuse with the plasma membrane, allowing for the immediate release of their cargo into the cytosol. Second, PENPs are frequently internalized via endocytic processes, including macropinocytosis, caveolin-mediated endocytosis, and clathrin-mediated endocytosis. These nonspecific mechanisms have been widely supported by experimental data across diverse plant sources and cellular models. For instance, in macrophages and intestinal stem cells, cytochalasin D treatment significantly reduced the uptake of grapefruit-derived PENPs, implicating macropinocytosis [70]. Similarly, chlorpromazine (an inhibitor of clathrin-mediated endocytosis) also markedly suppressed PENP internalization, confirming the involvement of this pathway [48]. Moreover, citrus-derived PENPs are enriched with membrane-associated proteins involved in transmembrane transport, vesicle trafficking, and intracellular shuttling, suggesting that these surface components may facilitate membrane recognition, vesicle formation, and intracellular transport during endocytosis [59].

**Figure 4:**
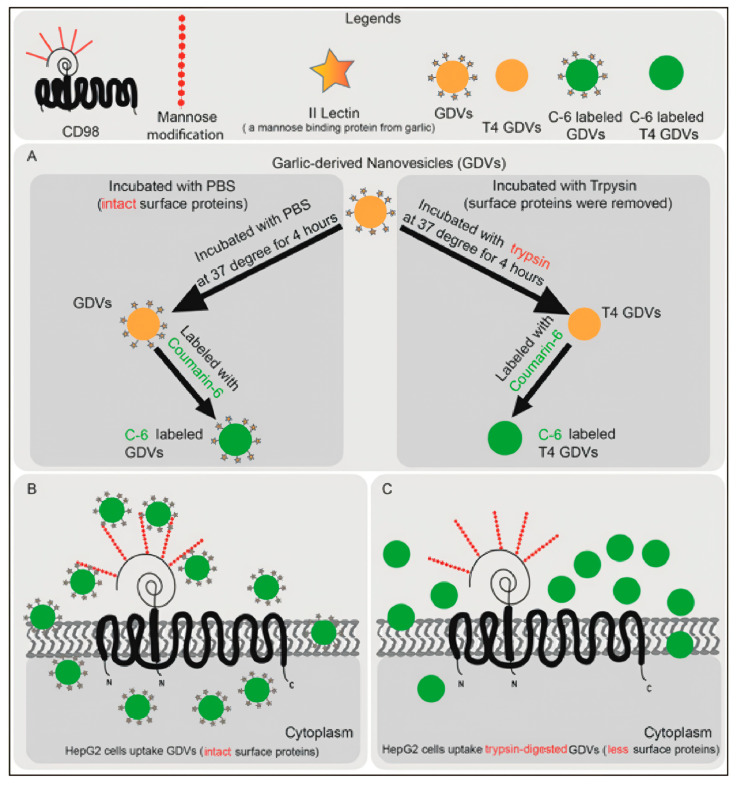
Schematic model of receptor-mediated endocytosis of garlic-derived PENPs via CD98 recognition. (**A**) Garlic-derived PENPs with intact surface proteins are labeled with coumarin-6 to enable visualization of cellular uptake. (**B**) Surface-expressed type II lectins on PENPs specifically recognize CD98 glycoproteins on HepG2 cell membranes, facilitating receptor-dependent endocytosis. (**C**) Removal of surface lectins by trypsin digestion disrupts CD98 recognition, leading to markedly reduced cellular internalization [61].

The third uptake mechanism is receptor-mediated endocytosis, driven by specific interactions between surface glycoproteins or proteins on PENPs and receptors on the target cell membrane. For example, type II lectins on garlic-derived PENPs have been shown to specifically bind CD98 glycoproteins on HepG2 cell membranes, thereby promoting receptor-dependent internalization, as schematically illustrated in Figure 4 [61].

PENP uptake efficiency is influenced by multiple variables and often displays clear time- and dose-dependent patterns. For example, blueberry-derived PENPs demonstrate dose-dependent internalization in endothelial cells whereas ginseng-derived PENPs exhibit progressive accumulation in mesenchymal stem cells over time [44]. Additionally, different cell types show varying preferences for uptake mechanisms, and ligand–receptor interactions between PENPs and target membranes play a critical role in determining internalization efficiency [107]. Although various endocytic inhibitors have been used to dissect these pathways, their limited specificity and potential off-target effects hinder the conclusive identification of dominant uptake routes [55].

Importantly, cellular internalization does not necessarily ensure effective intracellular delivery. Evidence suggests that internalized PENPs are frequently trafficked into the endosomal–lysosomal system, where their cargo may undergo degradation. For example, in a study, grapefruit-derived PENPs were observed to colocalize with lysosomes in HaCaT cells within six hours post uptake, suggesting that acidic lysosomal environments may inactivate their bioactive components [47]. Some PENPs are enriched in membrane-stabilizing lipids such as PA, which may help delay lysosomal fusion and improve cytosolic release efficiency [108]. Comprehensive studies on the intracellular trafficking dynamics and release kinetics of PENPs remain limited, posing a challenge to the rational design of PENP-based therapeutic delivery systems. Moreover, evidence regarding cellular uptake mechanisms and intracellular fate in humans is currently minimal, with most mechanistic insights derived from in vitro models or animal tissues, which warrants cautious extrapolation to clinical contexts.

### 6.2. Biodistribution Patterns and Delivery Strategy-Dependent Effects of PENPs

The biodistribution characteristics of PENPs play a critical role in determining their delivery efficiency and therapeutic specificity. Among the key factors influencing in vivo transport and organ accumulation are the route of administration and the vesicle lipid composition. PENPs from different plant origins exhibit diverse systemic stability and tissue distribution profiles, with their therapeutic efficacy being strongly dependent on delivery modality and structural attributes. In particular, lipid composition significantly affects biodistribution. For example, ginger-derived PENPs enriched in PA display strong intestinal adhesion and prolonged mucosal retention [109]. In contrast, grapefruit-derived PENPs, which are abundant in PC, preferentially accumulate in the liver [68]. These compositional differences likely influence interactions between PENPs and host cellular membranes or biological fluids, ultimately modulating their biodistribution patterns and organ-targeting behavior. However, current biodistribution data are derived predominantly from animal studies, with direct human evidence still scarce. In addition, most reports are of proof-of-concept tracer experiments rather than systematic pharmacokinetic or efficacy-oriented investigations. Therefore, distinguishing preliminary observations from true preclinical efficacy is essential for a rigorous evaluation of the translational potential of PENPs.

Different administration routes result in distinct pharmacokinetic behaviors and tissue distribution profiles, allowing for application across various disease contexts. Oral administration offers superior patient compliance and is well-suited for chronic conditions, particularly those related to gastrointestinal disorders. Multiple studies have demonstrated that orally delivered PENPs predominantly accumulate in the distal small intestine, cecum, and colon, where they are taken up by intestinal stem cells and macrophages. For instance, ginger-derived PENPs have been shown to reach the liver via the bloodstream, bypassing the lymphatic system. This supports their role in gut–liver axis regulation [98]. PENPs from grapefruit and ginseng administered via gastric gavage primarily localize within the gastrointestinal tract and demonstrate robust resistance to gastric acid and bile salts, indicating strong gastrointestinal stability [110]. Additionally, particle size and surface charge dynamics of PENPs vary in response to gastrointestinal conditions depending on the plant source, influencing their mucosal adhesion and intestinal retention potential [57].

Intravenous injection circumvents gastrointestinal degradation and first-pass metabolism, thereby enhancing systemic stability and promoting targeted organ delivery. DiR-based fluorescence imaging has revealed that ginseng-derived PENPs predominantly accumulate in the liver and spleen following intravenous or intraperitoneal administration. This persists in circulation beyond 48 h, indicating a relatively long systemic half-life [111]. Engineering modifications such as PEGylation have further improved pharmacokinetics. For example, PEGylated corn-derived PENPs have demonstrated extended circulation time and enhanced tumor-specific accumulation, validating the utility of surface engineering in optimizing delivery performance [112]. As a commonly employed route of administration in preclinical studies, intraperitoneal injection has also been demonstrated to facilitate the rapid systemic distribution of PENPs to highly perfused organs. Studies have demonstrated that grapefruit-derived PENPs, when administered intraperitoneally, predominantly accumulate in the liver, lungs, and spleen [47,70]. This distribution profile closely parallels that observed with intravenous injection, indicating the suitability of intraperitoneal delivery for evaluating the pharmacodynamic effects of PENPs in models of systemic inflammation and immune-mediated diseases.

Transdermal and intranasal routes represent non-invasive delivery alternatives that are especially promising for localized wound repair and brain-targeted therapy, respectively. Studies have shown that broccoli-derived PENPs can penetrate the stratum corneum and reach the dermal layer [113] whereas ginseng-derived PENPs facilitate the differentiation of stem cells into neural lineages in wound healing models. Intranasally delivered lipid-rich PENPs from grapefruit have been observed to accumulate in the lungs and brain, with prolonged fluorescent signals detected in the brain, suggesting their potential to cross the BBB [70,114]. A comparative summary of organ-specific distribution patterns, delivery characteristics, and corresponding therapeutic indications for each administration route is provided in Table 3.

## 7. Engineering Strategies and Therapeutic Applications of PENPs as Drug Delivery Platforms

### 7.1. Surface Engineering and Functionalization of PENPs

Surface engineering and the functionalization of PENPs constitute pivotal strategies to enhance their drug delivery efficiency, targeting specificity, and therapeutic performance. Rational surface modification enables improved drug loading capacity and facilitates selective accumulation in specific tissues or cell types, thereby optimizing treatment outcomes. Initial engineering efforts have primarily focused on passive targeting approaches. For example, surface conjugation with hydrophilic polymers such as PEG has been widely employed to prolong systemic circulation and enhance biocompatibility. PEGylated PENPs exhibit extended blood half-life and reduced clearance by the reticuloendothelial system (RES) [115]. However, while PEGylation improves systemic stability, it does not confer tissue specificity and may result in non-selective biodistribution, potentially diminishing therapeutic efficacy. In contrast, active targeting strategies leverage the conjugation of specific ligands such as peptides, antibodies, or small molecules onto the surface of PENPs to promote receptor-mediated cellular uptake. For instance, folic-acid-functionalized PENPs selectively bind to folate receptors overexpressed on certain cancer cells, thereby enhancing site-specific drug delivery and augmenting antitumor effects [116]. This targeted approach holds particular promise for cancer therapy and the treatment of diabetes-related complications, offering a robust platform for precision medicine [65]. Multiple studies have validated the therapeutic versatility of surface-engineered PENPs. Ginger-derived PENPs functionalized with folic acid have been developed as RNA delivery vehicles, significantly improving RNA stability and enhancing targeted uptake by tumor cells, thus elevating anticancer efficacy [117]. Similarly, lemon-derived PENPs modified with heparin have demonstrated the capacity to overcome drug resistance in tumor-bearing models, exhibiting potent therapeutic effects [118]. These findings underscore the potential of surface-engineered PENPs as a next-generation, biocompatible nanocarrier system for targeted therapeutic delivery.

### 7.2. Advantages of PENPs as Drug Carriers

PENPs offer several advantages as drug delivery vehicles owing to their natural origin, excellent biocompatibility, and low immunogenicity. As illustrated in Figure 1, PENPs possess a variety of bioactive molecular components and therapeutic functions, including metabolic regulation, immune modulation, antioxidant activity, and tissue repair, which support their engineering for targeted drug delivery applications. These inherent properties minimize the activation of the host immune system, rendering PENPs safer than synthetic nanocarriers such as liposomes [24]. For example, in a murine model of colitis, grape-derived PENPs elicited no significant immune rejection, demonstrating outstanding in vivo tolerability and consistent drug delivery performance [64]. Beyond their immunological safety, PENPs exhibit high cellular uptake efficiency and an intrinsic capacity to traverse biological barriers. In comparative studies, grapefruit-derived PENPs achieved intracellular uptake rates of over 80%, significantly exceeding that of conventional liposomes (~40%) in targeted cells [70]. Moreover, ginseng-derived PENPs were shown to effectively cross the BBB and accumulate in glioma tissue, underscoring their translational potential for central nervous system (CNS)-related diseases [31]. Collectively, these features confer PENPs with superior physiological stability, enhanced bioavailability, and improved tissue-targeting capabilities. These advantages establish PENPs as a highly promising class of nanocarriers for therapeutic applications in inflammatory diseases, cancer, and CNS disorders.

### 7.3. Drug Loading Techniques

To achieve efficient therapeutic delivery and enhance the bioavailability of therapeutic cargos, a variety of drug loading techniques have been developed for PENPs. Common strategies include co-incubation, sonication, electroporation, and freeze–thaw cycling. Each method differs in terms of operational complexity, drug compatibility, and encapsulation efficiency, and is selected based on specific physicochemical properties and delivery requirements. Co-incubation is the most commonly used and technically straightforward method, relying on the passive diffusion of drug molecules into PENPs by co-incubating them at an optimal temperature. This approach is particularly suitable for hydrophobic drugs. However, it exhibits limited efficiency for hydrophilic molecules and lacks precise control over encapsulation rates. For instance, in an attempt to encapsulate miRNA mimics such as *hsa-miR-340* into cherry-derived exosomes, co-incubation proved convenient but resulted in relatively low loading efficiency [119]. Sonication improves drug encapsulation by temporarily disrupting the lipid bilayer, thereby enhancing membrane permeability and facilitating drug entry. This technique is especially effective for small-molecule compounds and can significantly improve loading efficiency. Nevertheless, an excessive intensity or duration of sonication may compromise vesicle membrane integrity, potentially affecting stability and bioactivity. In one study, ginger-derived PENPs were loaded with doxorubicin (DOX) via sonication, achieving superior loading capacity compared to co-incubation while maintaining vesicle integrity and size uniformity [116]. Electroporation employs pulsed electric fields to induce transient pore formation in the vesicle membrane, allowing for the encapsulation of large or charged biomolecules. This method is particularly suitable for nucleic acid-based therapeutics such as siRNA and mRNA. In the brain-resident microglia of mice, siRNA was successfully loaded into extracellular vesicles via electroporation, resulting in the downregulation of the *BACE1* gene and demonstrating effective intracellular delivery [120]. Moreover, electroporation has been used to conjugate drug-loaded nanoparticles including gold nanoparticles (AuNPs) onto the surfaces of PENPs, enhancing targeting precision and delivery stability [121]. Freeze–thaw cycling involves the repeated freezing and thawing of PENPs, which transiently destabilizes the lipid membrane, enabling drug diffusion into the vesicles [122]. This mild technique is particularly advantageous for temperature-sensitive biologics such as enzymes or proteins as it preserves membrane structure and biological activity [90].

Each loading technique presents trade-offs between ease of operation, encapsulation yield, and vesicle stability. Therefore, method selection should be guided by the physicochemical properties of the drug and the intended therapeutic application. Recent studies suggest that combinatorial strategies including sonication followed by co-incubation can further enhance encapsulation efficiency and bioavailability [123]. These approaches offer promising avenues for the clinical translation of PENP-based drug delivery systems.

### 7.4. Drug Delivery Applications

As a natural nanocarrier system, PENPs exhibit outstanding biocompatibility, high cellular uptake efficiency, and inherent cross-species delivery capability. These properties have enabled their successful application across a wide range of disease models. Their unique lipid bilayer structure supports the encapsulation of both small-molecule compounds and macromolecular biologics, such as nucleic acids and proteins. This enables PENPs to hold substantial potential in the treatment of cancers, inflammatory disorders, autoimmune diseases, and metabolic syndromes [124,125].

**Figure 5:**
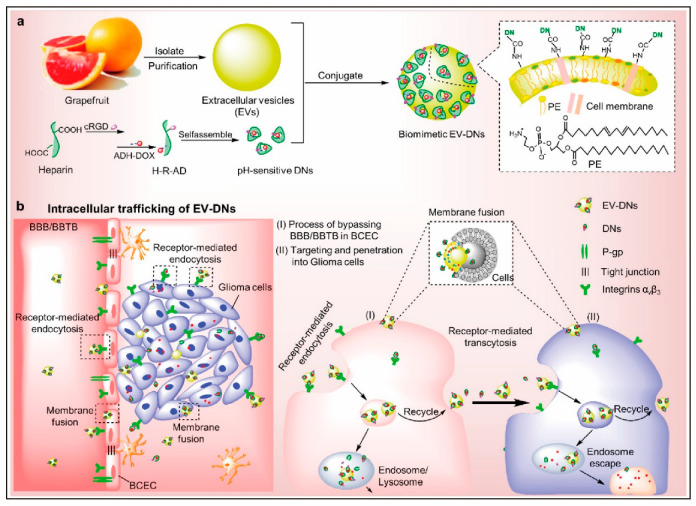
Grapefruit-derived extracellular-vesicle-based hybrid nanoparticles (EV-DNs) for glioma therapy. (**a**) The system combines the BBB-penetrating capacity of EVs with the drug-loading and targeting properties of pH-sensitive DNs; (**b**) The hybrid system enables efficient brain delivery and tumor-specific DOX release via αvβ3 integrin-mediated transcytosis and membrane fusion [126].

In oncology, PENPs have demonstrated enhanced tumor-targeting efficacy, particularly when surface-engineered with disease-specific ligands. This modification enables the precise delivery of chemotherapeutic agents while minimizing systemic toxicity. For example, folic-acid-modified ginger-derived PENPs selectively bind to folate receptors overexpressed on tumor cells. This targeted interaction results in increased siRNA delivery to the tumor site, enhanced antitumor activity, and reduced off-target cytotoxicity [117]. Moreover, DOX-loaded grapefruit-derived PENPs exhibited potent tumor-suppressive effects and were capable of penetrating the BBB in an in vivo model, indicating their promise in brain tumor therapy (Figure 5) [126].

Additionally, lemon-derived PENPs have been employed to deliver TRAIL pathway activators, inducing apoptosis in chronic myeloid leukemia (CML) cells and highlighting their potential utility in tumor immunotherapy [30].

In the field of inflammation and immune regulation, PENPs have shown significant therapeutic benefits. Grape-derived PENPs have effectively targeted lamina propria macrophages in murine colitis, delivering the immunosuppressive agent methotrexate and alleviating key inflammatory symptoms, including colon shortening, weight loss, and tissue injury [64]. Broccoli-derived PENPs were reported in a study to activate the AMPK signaling pathway and modulate cytokine profiles. These effects contributed to improved outcomes in both acute and chronic colitis, demonstrating strong tissue specificity and anti-inflammatory potential [49]. Furthermore, orange-derived PENPs have been explored as delivery systems for RNA vaccines, eliciting robust humoral and cellular immune responses. When administered intranasally or orally, they have successfully induced antigen-specific IgG production and T cell activation, underscoring their potential as mucosal vaccine platforms [127].

Collectively, these findings underscore the high targeting specificity, biological stability, and therapeutic versatility of PENPs across diverse disease contexts. With continued advances in drug loading and surface engineering technologies, PENPs are poised to evolve into next-generation, multifunctional, and low-toxicity nanocarriers, facilitating clinical translation in the treatment of major chronic and immune-mediated diseases.

## 8. Mechanisms of PENPs in the Treatment of Diabetes and Its Complications

### 8.1. Antioxidant and Anti-Inflammatory Activities

Oxidative stress and chronic low-grade inflammation are widely recognized as key pathogenic drivers in the onset and progression of T2DM [128]. The excessive production of reactive oxygen species (ROS) induces pancreatic β-cell apoptosis and disrupts insulin signaling, thereby exacerbating insulin resistance [129]. Simultaneously, elevated levels of pro-inflammatory cytokines such as tumor necrosis factor-alpha (TNF-α) and interleukin-6 (IL-6) further impair metabolic homeostasis [130]. This disruption perpetuates a pathological cycle of oxidative stress and inflammation.

**Figure 6:**
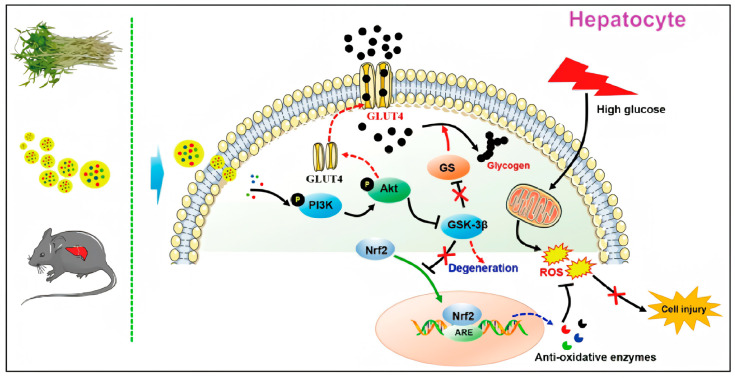
Proposed molecular mechanisms of mung-bean-sprout-derived PENPs in ameliorating diabetic hepatic injury. Mung-bean-sprout-derived PENPs improve diabetic conditions in HFD/STZ mice by modulating the PI3K/Akt/GLUT4/GSK-3β pathway, promoting glucose uptake and glycogen synthesis, and activating Nrf2-mediated antioxidant responses to alleviate oxidative stress-induced hepatocellular injury [131].

Recent studies have identified PENPs, which are enriched in antioxidative and immunomodulatory components, as promising natural nanotherapeutics capable of mitigating diabetes-induced oxidative and inflammatory damage. For instance, PENPs derived from mung bean sprouts were shown to activate the Nrf2/heme oxygenase-1 (HO-1) pathway, increase the activity of glutathione peroxidase (GSH-Px) and superoxide dismutase (SOD), and reduce hepatic malondialdehyde (MDA) levels, thereby attenuating lipid peroxidation and preserving liver function [131] (Figure 6).

Similarly, a dual-delivery system composed of *Rumex acetosa*-derived PENPs and selenium nanoparticles (TB-ELNs@SeNPs) significantly elevated SOD and GSH levels while suppressing TNF-α and interleukin-1 beta (IL-1β) expression in diabetic mice, via Nrf2-mediated signaling [8]. Ginger-derived nanoparticles (GDNPs) and their engineered platform (HMS/A@GE) have also been reported to reduce ROS accumulation, restore GSH content, and alleviate oxidative stress in hepatic and pancreatic tissues [65,132].

In addition to their antioxidative functions, PENPs exert potent anti-inflammatory effects. GDNPs have been shown to modulate the gut–liver axis, downregulate pro-inflammatory cytokines (TNF-α, IL-6), and upregulate the anti-inflammatory cytokine IL-10 in diabetic models [65]. Furthermore, a composite formulation incorporating PENPs from *Aloe vera*, *Curcuma longa* (turmeric), and *Gardenia jasminoides* into a chitosan membrane markedly reduced matrix metalloproteinases (MMP-2/9), TNF-α, and IL-6 while enhancing transforming growth factor-beta (TGF-β) expression in diabetic wound models. This formulation consequently accelerated tissue regeneration [133].

Notably, lemon-derived PENPs embedded in a biofunctional hydrogel have been shown to induce M2 macrophage polarization, characterized by the downregulation of inducible nitric oxide synthase (iNOS) and TNF-α, upregulation of arginase-1 (Arg-1) and IL-10, and suppression of nuclear factor-kappa B (NF-κB) signaling. These changes ultimately remodel the inflammatory microenvironment in diabetic wounds [134]. Additionally, garlic-derived PENPs have been reported to interact with outer membrane vesicles released by *Akkermansia muciniphila*, strengthening intestinal tight junctions and reducing circulating pro-inflammatory cytokine levels [40].

Collectively, these findings suggest that PENPs orchestrate antioxidant and immunoregulatory responses through multiple signaling axes, including the Nrf2/HO-1, NF-κB, and aryl hydrocarbon receptor (AhR) pathways. These mechanisms provide a promising foundation for the development of multi-targeted therapeutic strategies for T2DM and its complications. However, the current evidence remains largely restricted to preclinical studies, and further investigation is required to assess long-term efficacy and clinical safety.

### 8.2. Modulation of Gut Microbiota and Immune Homeostasis

The dysregulation of the gut microbiota is closely associated with the pathogenesis of T2DM [135]. Diets high in fat and sugar typically reduce the abundance of beneficial microbes, diminish short-chain fatty acid (SCFA) production, and impair intestinal barrier integrity [136]. These changes facilitate the translocation of endotoxins such as lipopolysaccharide into the systemic circulation, thereby triggering chronic inflammation and aggravating insulin resistance [137]. Accordingly, the restoration of the gut microbiota–immune axis has emerged as a promising therapeutic approach in diabetes management [138].

As multifunctional natural nanocarriers, PENPs have demonstrated significant potential in modulating gut microbial composition, reinforcing epithelial barrier function, and reestablishing immune homeostasis. In diabetic mouse models, a dual-delivery system comprising *Rumex acetosa*-derived PENPs and selenium nanoparticles (TB-ELNs@SeNPs) significantly enriched SCFA-producing bacterial taxa such as *Dubosiella* and *Lachnospiraceae* while concurrently suppressing pathogenic genera including *Helicobacter*. These microbial shifts were accompanied by increased fecal SCFA concentrations and improved metabolic outcomes [139].

Additional studies reported that the ginger-derived HMS/A@GE platform promoted the expansion of *Lactobacillus* species, regulated intestinal tryptophan metabolism, and elevated levels of AhR ligands such as indole and indole-3-acetic acid (IAA). The activation of the AhR-IL-22 axis enhanced the expression of tight junction proteins, improved gut barrier integrity, suppressed local inflammation, and ultimately ameliorated glucose metabolism [132].

Likewise, PENPs derived from orange peel were shown to modulate bile acid metabolism and promote SCFA production in *db*/*db* mice, leading to microbiota rebalancing, reduced systemic inflammation, and improvements in both insulin sensitivity and lipid metabolism [41]. Garlic-derived PENPs were found to be internalized by *Akkermansia muciniphila*, triggering the release of outer membrane vesicles enriched in immunomodulatory components. These OMVs upregulated the expression of tight junction proteins such as zonula occludens-1 (ZO-1) and occludin while downregulating inflammatory cytokines including IL-1β and TNF-α, thereby promoting systemic immune equilibrium [40].

**Figure 7:**
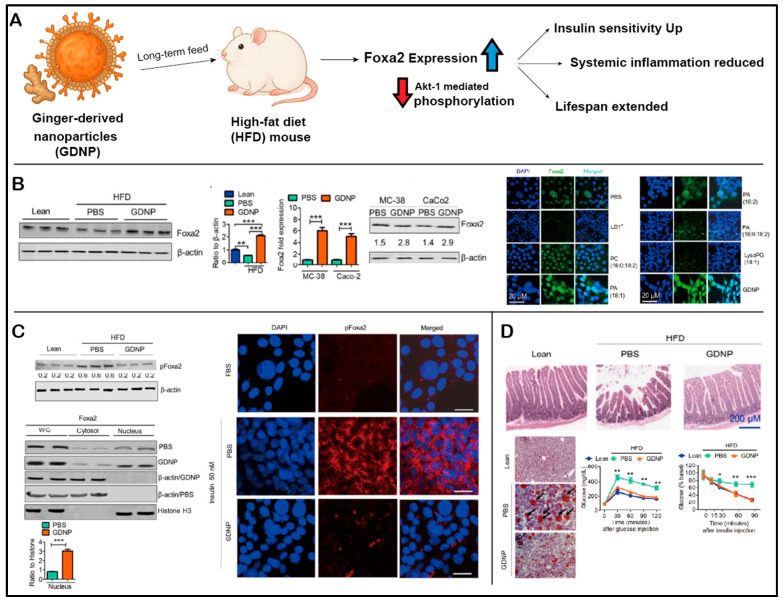
GDNPs activate Foxa2 signaling and improve metabolic health in HFD-fed mice. (**A**) Schematic representation of the proposed mechanism by which GDNPs modulate Foxa2 signaling in HFD-fed mice. (**B**) GDNPs treatment upregulates total Foxa2 expression in both cultured cells and small intestinal tissues. (**C**) GDNPs inhibit Foxa2 phosphorylation and reduce phosphorylated Foxa2 (pFoxa2) levels in cell culture and small intestinal tissues of lean and HFD mice. (**D**) GDNPs alleviate HFD-induced small intestinal damage, reduce liver weight and fat accumulation, and protect against glucose intolerance and insulin resistance [65]. * *p* < 0.05, ** *p* < 0.01, *** *p* < 0.001.

Moreover, ginger-derived PENPs (GDNPs) were reported to activate the Forkhead box A2 (Foxa2) signaling pathway, enhance the expression of tight junction proteins in the small intestine, and alleviate inflammation through reductions in ROS (Figure 7) [65]. The co-delivery of molecular hydrogen (H_2_) further enhanced this effect, demonstrating synergistic activity in microbiota restoration and gut–immune axis modulation [65,132].

In summary, PENPs exert multifaceted regulatory effects on diabetes-associated gut dysbiosis by promoting probiotic colonization, elevating SCFA and tryptophan-derived metabolite production, reinforcing epithelial barrier architecture, and suppressing inflammatory signaling. These integrated actions highlight the promise of PENPs as microbiota-targeted nanotherapeutics for immune metabolic diseases such as T2DM. Nevertheless, despite these therapeutic benefits in diabetic models, the long-term safety of PENP-mediated microbiota modulation remains poorly defined. In particular, potential risks such as unintended alterations in microbial composition, persistence or reversibility after treatment withdrawal, and interactions with dietary factors or antibiotics have not been systematically investigated. To address these unresolved issues, future studies should incorporate longitudinal multi-omics approaches and dose–response designs.

### 8.3. Regulation of Glucose Metabolism and Insulin Signaling Pathways

The pathogenesis of T2DM is primarily characterized by insufficient insulin secretion and peripheral insulin resistance, resulting in impaired glucose uptake and increased hepatic gluconeogenesis [140]. Restoring insulin signaling and improving systemic glucose homeostasis therefore remain essential therapeutic goals [141].

An increasing body of evidence suggests that PENPs exert significant regulatory effects on glucose metabolism and insulin signaling pathways. In high-fat diet and streptozotocin (HFD/STZ)-induced diabetic mice, mung-bean-sprout-derived PENPs significantly lowered fasting blood glucose, total cholesterol, and triglyceride levels. These improvements were accompanied by enhanced performance in oral glucose tolerance tests (OGTTs) and insulin tolerance tests (ITTs), reduced hepatic lipid deposition, and improved islet morphology and functional preservation [131].

A dual-delivery formulation composed of *Rumex acetosa*-derived PENPs and selenium nanoparticles (TB-ELNs@SeNPs) activated both the phosphoinositide 3-kinase (PI3K)/protein kinase B (Akt) and AMPK pathways. This dual activation led to the downregulation of gluconeogenic enzymes (PEPCK and G6Pase) and lipogenic regulators (SREBP-1c and FAS), thereby improving insulin sensitivity, suppressing hepatic glucose production, and restoring metabolic homeostasis [139].

Ginger-derived PENPs were shown to enhance the intestinal expression of glucose transporter 2 and facilitate glucose translocation by upregulating the Foxa2 signaling axis, effectively mitigating HFD-induced insulin resistance [65]. In a composite HMS/A@GE platform, the co-delivery of PENPs with molecular H_2_ further augmented PI3K/Akt signaling activity, enhanced glucose uptake in hepatic and muscular tissues, and significantly reduced the homeostatic model assessment of insulin resistance (HOMA-IR) index [132].

Similarly, orange-peel-derived PENPs restored the expression of key metabolic regulators including peroxisome proliferator-activated receptor gamma coactivator 1-alpha (PGC-1α), insulin receptor substrate-1 (IRS-1), and GLUT4 in *db*/*db* mice. These changes were associated with decreased hepatic steatosis, reduced inflammatory cytokine production, and improved insulin signaling fidelity [41]. Garlic-derived PENPs, through their interaction with *Akkermansia muciniphila*, stimulated GLP-1 secretion in the gut. This activation of the GLP-1 receptor (GLP-1R) cascade led to the upregulation of IRS1/2 and Akt, effectively reinforcing gut–pancreas axis communication and reversing HFD-induced insulin signaling disruption [40].

Collectively, PENPs orchestrate metabolic reprogramming through multiple synergistic pathways. On one hand, they activate PI3K/Akt and AMPK signaling to inhibit gluconeogenesis and lipogenesis while promoting glucose uptake and utilization. On the other hand, they enhance the expression of glucose transporters and stimulate GLP-1 secretion, thereby indirectly improving insulin sensitivity and islet function. These therapeutic effects are frequently accompanied by reductions in systemic inflammation and oxidative stress, highlighting the promise of PENPs in mediating multi-targeted metabolic regulation. Future studies should focus on elucidating the upstream molecular targets and signaling networks responsible for these effects to support the clinical translation of PENP-based metabolic interventions.

### 8.4. Promotion of Angiogenesis and Metabolic Reprogramming

Diabetic microangiopathy and ischemic tissue injury, particularly impaired wound healing, are closely associated with defective angiogenesis and local metabolic dysfunction [142]. In recent years, PENPs have garnered attention for their ability to promote neovascularization and induce metabolic reprogramming, thereby offering novel strategies for tissue regeneration through the modulation of cellular energy metabolism, the immune microenvironment, and key signaling cascades [57].

**Figure 8:**
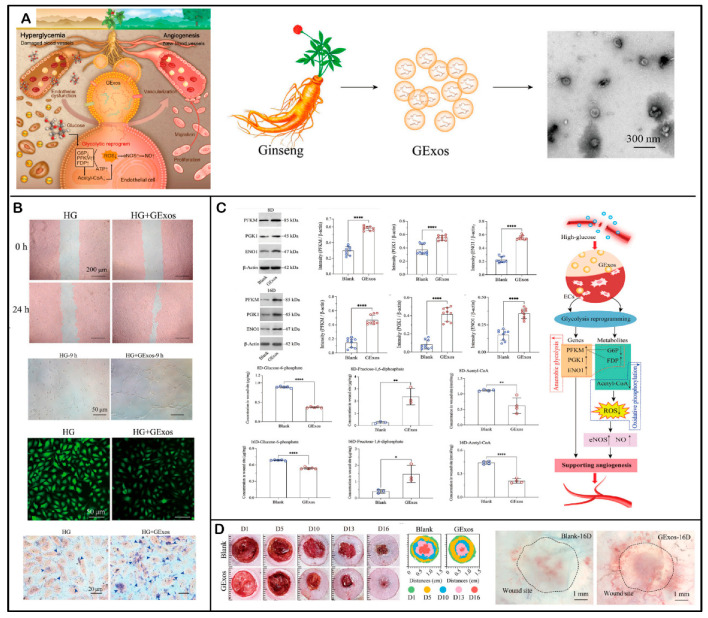
GExos promote angiogenesis and wound healing via glycolytic reprogramming. (**A**) Schematic illustration of GExos promoting angiogenic activity in endothelial cells under high-glucose conditions via glycolytic reprogramming, alongside the isolation and TEM characterization of GExos derived from ginseng. (**B**) GExos enhance endothelial cell migration and tubule formation in high-glucose culture and modulate intracellular ROS levels and oxidative phosphorylation activity. (**C**) Western blot and metabolic analyses demonstrating the therapeutic mechanism of GExos in promoting glycolysis-dependent angiogenesis in diabetic conditions. (**D**) GExos facilitate microvascular network formation and accelerate wound healing in a diabetic mouse model [143]. * *p* < 0.05, ** *p* < 0.01, **** *p* < 0.0001.

Ginseng-derived PENPs are enriched with bioactive constituents, notably specific miRNAs and ginsenosides Rg1, Re, and Rb1. In a study, in high-glucose-stimulated human umbilical vein endothelial cells (HUVECs), these PENPs significantly upregulated glycolytic enzymes including phosphofructokinase muscle-type (PFKM), phosphoglycerate kinase 1 (PGK1), and enolase 1 (ENO1). Consequently, anaerobic glycolysis was upregulated, leading to an increase in intracellular ATP levels and suppression of mitochondrial oxidative phosphorylation (Figure 8). These metabolic reprogramming events improved endothelial energy metabolism and vascular functionality [143]. In a *db*/*db* diabetic mouse wound model, ginseng-derived PENPs markedly elevated the expression of vascular endothelial growth factor (VEGF) and CD31 at the wound site, increasing microvessel density by 2.72-fold compared to controls, underscoring their potent proangiogenic effect in vivo [143].

Lemon-derived PENPs embedded within functionalized hydrogels promoted M2 macrophage polarization in diabetic wounds, enhanced endothelial nitric oxide synthase (eNOS) expression and nitric oxide (NO) production, and downregulated pro-inflammatory markers such as inducible nitric oxide synthase (iNOS) and tumor necrosis factor-alpha (TNF-α) in a study. These immunomodulatory effects contributed to improved local immune homeostasis and endothelial function [134]. Mechanistic investigations further demonstrated that lemon-derived PENPs inhibited NF-κB signaling, thereby reducing inflammation and promoting tissue regeneration, establishing a pro-repair immune-metabolic environment [134].

Taken together, these findings highlight that PENPs support diabetic tissue repair through multifactorial mechanisms involving metabolic rewiring, immunomodulation, and angiogenic activation. By enhancing glycolytic flux and energy availability, PENPs promote endothelial cell proliferation and migration. Simultaneously, they modulate macrophage phenotypes and inflammatory pathways to generate a regenerative niche conducive to neovascularization and wound healing.

In summary, PENPs represent a promising nanotherapeutic modality for the management of diabetic vascular complications, chronic non-healing wounds, and ischemia-associated disorders. Their integration into biomaterial-based delivery systems may further enhance targeting precision, therapeutic responsiveness, and tissue-specific regeneration, paving the way for next-generation regenerative nanomedicine.

## 9. Application of PENPs in the Treatment of Diabetes and Its Complications

Despite recent advances in conventional therapies for diabetes, the long-term use of hypoglycemic agents remains associated with several limitations, including adverse side effects, drug resistance, and the disruption of metabolic homeostasis. As a result, natural plant-derived bioactive constituents have attracted growing attention in biomedical research due to their multi-targeted mechanisms, improved safety profiles, and intrinsic biocompatibility. The integration of nanotechnology into the delivery of these phytochemicals has been actively investigated as a strategy to enhance their therapeutic efficacy and overcome barriers such as poor solubility, low bioavailability, and instability in physiological environments [144,145].

For example, pterostilbene-loaded nanoparticles significantly reduced blood glucose levels following intravenous administration in diabetic rat models [146]. Similarly, curcumin nanoparticles prepared using an optimized emulsification–diffusion–evaporation method improved both fasting blood glucose and glycated hemoglobin levels [147]. In another study, the combined application of curcumin nanoparticles and aged garlic extract ameliorated diabetic cardiomyopathy by reducing myocardial inflammation, attenuating fibrosis, and lowering the risk of cardiovascular complications [27].

Against this backdrop, PENPs have emerged as a novel and promising platform in the treatment of diabetes and its associated complications. Unlike synthetic nanocarriers, PENPs possess a dual-functional role, acting both as endogenous nanocarriers and as intrinsic bioactive agents [108]. Extensive preclinical research has shown that PENPs can significantly improve the solubility, metabolic stability, and targeted tissue delivery of loaded phytochemicals without requiring synthetic surfactants or stabilizers [57]. Furthermore, their native bioactive components, such as antioxidant enzymes, polyphenols, and regulatory miRNAs, endow PENPs with intrinsic biological activity. These components enable PENPs to directly modulate key pathological processes including oxidative stress and inflammation [148,149]. This section summarizes the latest advances in preclinical studies exploring the use of PENPs for the treatment of diabetes and its complications, including hepatic lipid dysregulation and chronic diabetic wound healing.

### 9.1. Antihyperglycemic Effects

In high-fat diet/streptozotocin (HFD/STZ)-induced T2DM mouse models, mung-bean-sprout-derived PENPs have demonstrated pronounced antihyperglycemic effects. These PENPs significantly lowered fasting blood glucose levels, improved glucose tolerance in OGTT, and enhanced insulin sensitivity in ITT. Furthermore, treatment with mung-bean-sprout-derived PENPs effectively alleviated hepatic lipid accumulation and increased the islet β-cell area, indicating improved pancreatic function and β-cell preservation [131].

Similarly, a dual-carrier exosomal platform composed of Rumex acetosa-derived PENPs and selenium nanoparticles (TB-ELNs@SeNPs) exhibited robust glycemic control in T2DM mice. This hybrid system significantly reduced fasting blood glucose levels, improved glucose tolerance, attenuated dysregulated lipid metabolism, and broadly ameliorated metabolic disturbances in diabetic animals [139].

Collectively, these findings underscore the potential of PENPs to restore glucose homeostasis and enhance pancreatic islet function in diabetic models. Nevertheless, the precise molecular mechanisms underlying these effects remain to be fully elucidated and warrant further investigation.

### 9.2. Amelioration of Hepatic Lipid Metabolism Dysregulation

Non-alcoholic fatty liver disease and T2DM frequently coexist, sharing key pathophysiological features such as hepatic lipid accumulation and insulin resistance [150]. A self-perpetuating cycle between hepatic steatosis and impaired insulin signaling further exacerbates metabolic dysfunction [151]. Thus, targeting hepatic lipid metabolism represents a critical therapeutic strategy for improving diabetic outcomes.

**Figure 9:**
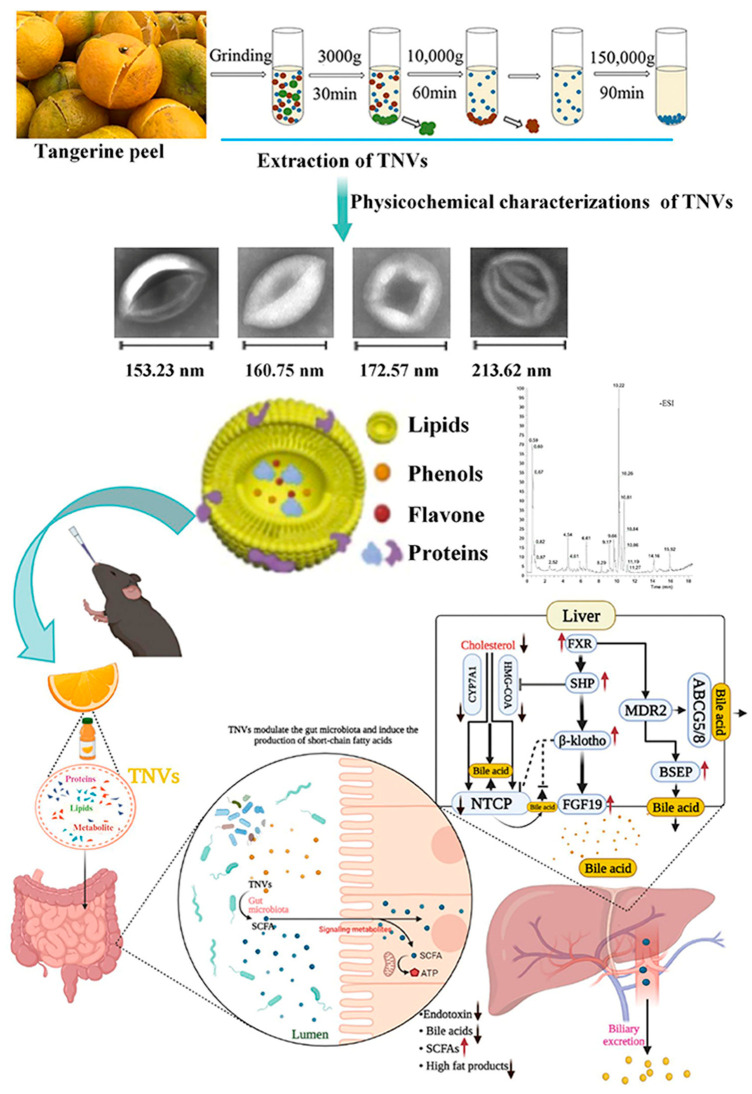
Overview of the isolation, characterization, and gut–liver regulatory effects of TNVs. TNVs were extracted via differential centrifugation and found to contain lipids, phenols, flavones, and proteins. Upon oral administration, TNVs modulated gut microbiota and promoted SCFA production, which in turn influenced bile acid metabolism and FXR-FGF19 signaling in the liver, contributing to metabolic improvement. Adapted from ref [41], 2024, under CC BY-NC 3.0 license.

In *db*/*db* diabetic mouse models, tangerine peel-derived nanovesicles (TNVs) significantly reduced hepatic lipid droplet area and decreased serum total cholesterol and triglyceride levels. Additionally, TNVs restored intestinal mucosal barrier integrity and reshaped gut microbiota composition, thereby alleviating hepatic lipid dysregulation through the modulation of the gut–liver axis [41] (Figure 9). These findings underscore the therapeutic potential of TNVs in modulating intestinal–hepatic crosstalk and provide a novel strategy for treating metabolism-associated fatty liver disease.

**Figure 10:**
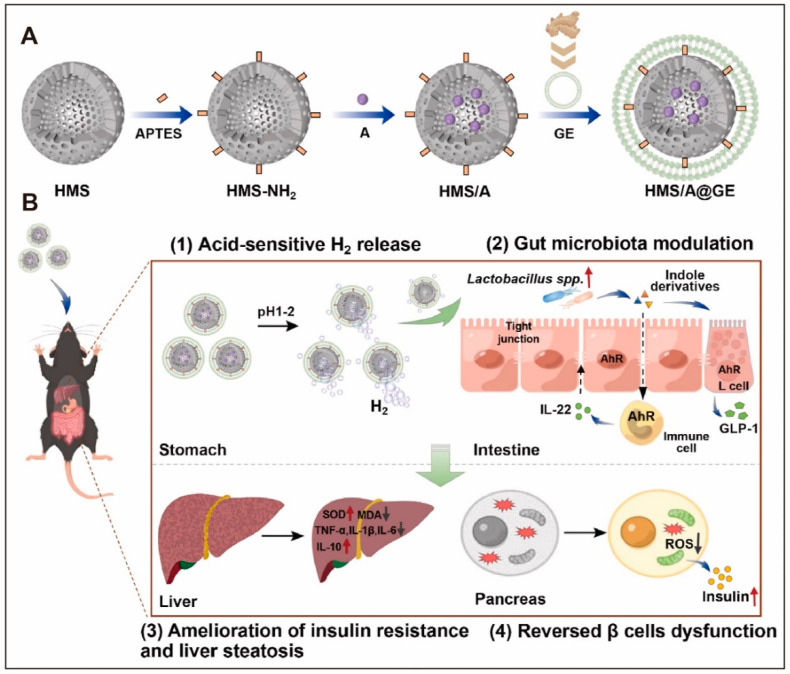
Illustration of construction and therapeutic mechanism of a biomimetic-acid-responsive nano hydrogen producer (HMS/A@GE). (**A**) Schematic of the synthesis procedure of HMS/A@GE. (**B**) Schematic depicting that HMS/A@GE modulates the gut microbiota composition and metabolites and exerts antioxidant and anti-inflammatory effects for collaboratively improving intestinal-barrier function, glucose dysmetabolism, and liver steatosis [132].

Furthermore, the HMS/A@GE platform, which consists of a borane and ginger composite system, exhibited potent hepatoprotective effects in HFD-induced T2DM models. By co-delivering ginger-derived PENPs and molecular H_2_, HMS/A@GE significantly reduced hepatic MDA levels and enhanced the expression of antioxidant enzymes such as GSH and SOD, thereby mitigating lipid peroxidation [132] (Figure 10). Concurrently, serum ALT and AST levels were markedly decreased, indicating the attenuation of hepatocellular inflammation and apoptosis. These data suggest the potential of this composite nanoplatform in alleviating diabetes-related liver dysfunction.

Collectively, PENPs exert multifaceted regulatory effects on hepatic lipid metabolism in diabetes by modulating lipid synthesis and degradation pathways, reducing oxidative stress, restoring gut barrier function, and rebalancing microbiota composition. Future studies should focus on elucidating the underlying signaling pathways, transcriptional regulators, and lipid droplet metabolism networks involved in PENP-mediated hepatic protection to facilitate clinical translation.

### 9.3. Therapeutic Applications of PENPs in Diabetic Wound Healing

Chronic, non-healing wounds are a prevalent and challenging complication of DM, primarily driven by sustained hypoxia, chronic inflammation, impaired angiogenesis, and reduced cellular migration [152]. In recent years, PENPs have attracted increasing attention for their promising role in accelerating diabetic wound healing.

Studies on ginseng-derived exosomes (GExos) have revealed that these nanovesicles are enriched with functional miRNAs and active ginsenosides, notably Rg1, Re, and Rb1. GExos were shown to enhance glycolytic metabolism, elevate intracellular ATP production, and activate key regenerative pathways including PI3K/Akt and eNOS signaling. In a full-thickness skin wound model using *db*/*db* diabetic mice, GExo treatment resulted in a 2.72-fold increase in capillary density and a 1.66-to-2.32-fold upregulation of CD31 protein expression. These effects significantly accelerated wound closure, demonstrating potent proangiogenic and tissue-regenerative capacities [143].

**Figure 11:**
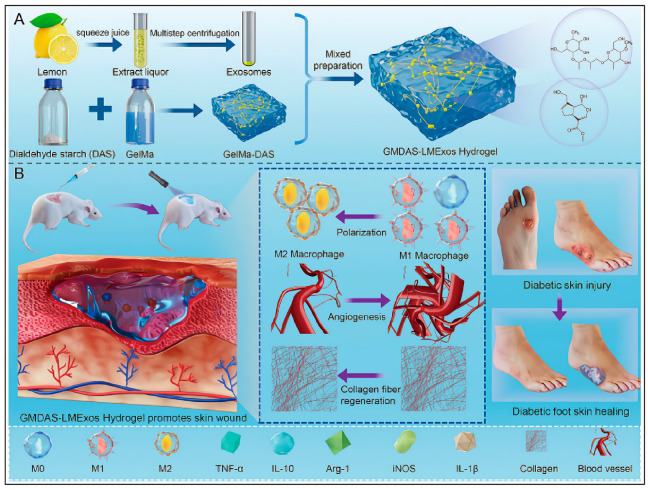
GelMA/DAS/Exo hydrogel was used as a dressing for diabetic wound healing. (**A**) Preparation of the lemon exosome hydrogel. (**B**) The lemon exosome hydrogel promoted diabetic wound healing by regulating macrophage polarization (M0, M1, and M2 defined as unpolarized, pro-inflammatory, and anti-inflammatory macrophages) and promoting fibroblast and vascular endothelial cell proliferation [134].

Similarly, lemon-derived PENPs incorporated into a functionalized hydrogel matrix significantly modulated the diabetic wound microenvironment. This hydrogel system promoted M2 macrophage polarization, upregulated IL-10 expression, and inhibited iNOS expression, thereby reducing local inflammation and improving tissue perfusion. These immunomodulatory and angiogenic effects facilitated granulation tissue formation and neovascularization. Furthermore, the hydrogel exhibited favorable bioadhesive and sustained-release properties, allowing prolonged PENP activity at the wound site [134] (Figure 11).

In another study, a composite chitosan nanomembrane loaded with PENPs derived from aloe (*Aloe barbadensis*), turmeric (*Zingiber officinale*), and neem (*Azadirachta indica*) was applied in a diabetic mouse skin injury model. This biomaterial significantly reduced wound area, suppressed MMP-2/9 expression, and increased TGF-β levels, thereby enhancing collagen deposition and keratinocyte proliferation, which collectively promoted rapid re-epithelialization and wound closure [133].

Collectively, these findings suggest that PENPs accelerate diabetic wound healing through multiple synergistic mechanisms, including the promotion of angiogenesis, immune microenvironment remodeling, and the stimulation of cellular migration and tissue regeneration. Future research should focus on the development of targeted PENP-based hydrogels, stimuli-responsive delivery systems, and combination therapies with stem cells or bioceramic scaffolds to establish comprehensive and integrative strategies for managing chronic diabetic wounds.

Although preclinical studies of PENPs in diabetes have yielded promising results, substantial methodological limitations persist. Most investigations rely on fixed dosing regimens without systematic evaluations of dose–response relationships or pharmacokinetic parameters. Evidence regarding long-term safety, in vivo stability, biodistribution, and the durability of therapeutic effects after single administration remains scarce, and findings are frequently restricted to single animal models with limited reproducibility. Future research should prioritize establishing dose–response relationships, elucidating pharmacokinetics and biodistribution, assessing long-term safety, and validating efficacy across models to advance clinical translation.

Furthermore, extracellular vesicles from mammalian sources, such as adipose tissue, hepatocytes, and mesenchymal stem cells, have also exhibited antioxidant, immunomodulatory, and metabolic regulatory effects in diabetic models [153].This indicates that some PENP effects are likely generalizable through conserved EV-mediated mechanisms [154]. Conversely, functions driven by plant-specific lipids and phytochemicals may be unique to PENPs, and further comparative studies are required to define the extent of conservation versus specificity.

## 10. Current Challenges and Future Directions

### 10.1. Challenges and Optimization Strategies in the Development of PENPs

PENPs have emerged as highly promising natural nanocarriers for the treatment of diabetes due to their favorable biocompatibility, multi-component bioactivity, and widespread availability. However, several critical challenges continue to hinder their development. Firstly, the lack of standardized protocols for isolation and purification remains a major obstacle. Common methods such as ultracentrifugation, density-gradient centrifugation, and ultrafiltration often suffer from inconsistent operational parameters, low yield, and significant batch-to-batch variability. These limitations impede reproducibility and restrict scalability for industrial application. Furthermore, the physiological status of the plant species, tissue types, harvest time, and processing methods can introduce substantial heterogeneity in particle size, composition, and biofunctionality. Secondly, the natural constituents of PENPs, including microRNAs, polyphenols, and proteins, exhibit complex pharmacological activities. Potential synergistic or antagonistic interactions between these bioactive components and the therapeutic cargos may influence the ultimate efficacy. Yet, comprehensive studies on these interaction mechanisms are still lacking, introducing uncertainty for clinical translation. Thirdly, insufficient targeting capability remains a bottleneck for effective delivery. Due to the limited presence of functional surface proteins on native PENPs, nonspecific biodistribution often occurs in vivo. Although exogenous ligand modification has been explored to enhance tissue-specific targeting, concerns regarding immunogenicity have not been adequately addressed, particularly in diabetic populations where immune responses are often dysregulated. Moreover, a unified nomenclature, classification criteria, and quality control metrics for PENPs have not been established. Systematic toxicological evaluation, especially for non-oral routes such as intravenous administration, is also lacking. Although a few preliminary clinical trials have investigated the oral safety of PENPs, the field remains in its infancy.

### 10.2. Challenges in the Treatment of Diabetes of PENPs

Despite the remarkable potential of PENPs as natural nanodelivery systems, their application in the treatment of diabetes and its complications remains at an early stage. To facilitate effective clinical translation, several disease-specific challenges must be addressed.

Diabetes is characterized by a complex pathophysiology that involves multiple target organs, including the liver, pancreas, kidneys, and skin. However, current studies have not systematically evaluated the biodistribution, targeting specificity, or delivery efficiency of PENPs in these tissues. The diabetic microenvironment is often dominated by chronic hyperglycemia and low-grade inflammation. These pathological conditions may compromise the structural stability and functional integrity of PENPs, thereby diminishing their therapeutic potential.

Another critical issue concerns the interaction of PENPs with conventional antidiabetic medications. There is currently insufficient evidence to determine whether PENPs are compatible or synergistic with mainstream therapies, such as insulin analogs, metformin, GLP-1R agonists, or sodium–glucose co-transporter 2 (SGLT2) inhibitors. Moreover, diabetic patients exhibit considerable heterogeneity in metabolic phenotypes, underscoring the urgent need for predictive biomarkers and therapeutic response criteria to identify suitable patient populations. Without individualized selection strategies, future clinical trials may face challenges in efficacy evaluation and safety assessment.

### 10.3. Barriers in the Treatment of Diabetic Complications

The treatment of diabetic complications frequently involves overcoming multiple physiological barriers, including the skin barrier in chronic non-healing wounds, the BBB in diabetic encephalopathy, and the glomerular filtration barrier in diabetic nephropathy. Although some studies suggest that PENPs are capable of penetrating these barriers, their performance under chronic pathological conditions remains largely unverified. For instance, it is still unclear whether PENPs can effectively access fibrotic or ischemic tissues and maintain functional activity within injured microenvironments such as diabetic wounds or fibrotic renal tissues.

Moreover, most existing research has been conducted in acute or short-term animal models. However, long-term studies assessing the stability, tolerability, and immune responses of PENPs during prolonged disease progression are still lacking. This absence of chronic exposure data limits the comprehensive evaluation of safety and reduces the translational relevance of PENPs for treating chronic diabetic complications.

In addition to wound healing and diabetic nephropathy, diabetes is commonly associated with severe complications such as cardiovascular disease, diabetic retinopathy, and both central and peripheral neuropathies. However, investigations into the therapeutic potential of PENPs for these conditions are extremely limited. For example, no study has systematically evaluated the role of PENPs in modulating cardiac inflammation, endothelial dysfunction, or vascular remodeling, all of which are recognized as key mechanisms in the development of diabetic cardiovascular complications. Similarly, it remains poorly understood whether PENPs can effectively cross the blood–retina barrier, maintain retinal vascular integrity, or alleviate oxidative stress and inflammatory responses within the retinal microenvironment.

While preliminary evidence suggests the neuroprotective potential of PENPs, their specific role in diabetic neuropathy has not been directly demonstrated. Research into their mechanisms of action in regulating neuroinflammation, axonal injury, Schwann cell function, or neurotrophic factor signaling remains scarce. These knowledge gaps highlight the pressing need for systematic investigations into the use of PENPs in the management of high-burden diabetic complications. Addressing these issues may enable the development of multi-targeted therapeutic strategies that simultaneously alleviate metabolic dysregulation and organ-specific damage.

### 10.4. Translational Barriers and Application Limitations

Despite promising preclinical data, the clinical translation of PENPs is limited by key challenges, notably the lack of standardized experimental models. Differences in the use of high-fat diet combined with streptozotocin (HFD/STZ) models, *db*/*db* mice, and Zucker diabetic fatty rats result in variable outcomes that hinder cross-study comparability. Furthermore, although oral administration is considered a practical and non-invasive delivery route, diabetic patients often present with gastrointestinal dysfunctions such as delayed gastric emptying and impaired nutrient absorption. These conditions may substantially compromise the bioavailability and pharmacokinetic profiles of orally administered PENPs.

Additionally, toxicological assessments remain insufficient. Existing studies predominantly focus on acute toxicity or short-term administration. Comprehensive evaluations of long-term safety, immunogenicity, and potential off-target effects under conditions of chronic or repeated exposure are still lacking. This is particularly critical for vulnerable patient populations, including those with advanced diabetes, impaired renal function, or compromised immune systems.

Moreover, the absence of standardized GMP protocols for the large-scale production, purification, and quality control of PENPs represents a significant bottleneck for clinical translation and industrial scalability.

### 10.5. Future Research Directions

PENPs, as natural nanocarrier platforms, have demonstrated excellent biocompatibility and multi-target regulatory potential in the treatment of diabetes and its complications. However, the development of PENPs, from platform construction to clinical application, continues to face multiple challenges, including source variability, compositional complexity, limited targeting efficiency, and the need to maintain structural stability and functional integrity within hyperglycemic and inflammatory microenvironments. To achieve efficient delivery and precise therapy, future research should focus on standardizing production processes, elucidating biological mechanisms, and developing organ-specific delivery strategies.

Given that diabetes is a systemic and chronic metabolic disorder characterized by complex clinical phenotypes and a narrow therapeutic window, with complications affecting the cardiovascular system, kidneys, nervous system, retina, and skin, the design and efficacy evaluation of nanomedicine systems is particularly demanding. Therefore, it is necessary to develop delivery platforms based on PENPs that possess enhanced targeting specificity, multimodal regulatory capacity, and adaptability to pathological tissues. A comprehensive progression from mechanistic understanding to precise intervention across multiple pathways should be pursued.

A key research priority is the systematic elucidation of the molecular mechanisms by which PENPs exert their therapeutic effects. The application of multi-omics technologies, including transcriptomics, proteomics, lipidomics, and metabolomics, should be employed to systematically investigate the regulatory functions of PENPs in diabetes-related target cells. Particular emphasis should be placed on their involvement in modulating inflammatory responses, oxidative stress, insulin signaling, and metabolic homeostasis. Further clarification of the functional roles and synergistic interactions of endogenous bioactive components within PENPs will facilitate the construction of a regulatory network linking bioactive constituents, molecular targets, and disease phenotypes.

Equally important is the enhancement of tissue and organ specificity for PENP-based delivery. Owing to their broad biodistribution in vivo, enhancing delivery specificity to diabetic target organs such as the pancreas, liver, kidneys, myocardium, retina, and nervous system is essential. Approaches such as surface functionalization, the enrichment of natural adhesion molecules, and the engineering of ligand modifications may significantly improve their accumulation within specific tissues and enhance cellular uptake, thereby strengthening local therapeutic efficacy.

To ensure therapeutic effectiveness under the adverse microenvironments characteristic of diabetic complications, including acidic pH, hypoxia, and oxidative stress, future efforts should develop hybrid delivery platforms. By integrating PENPs with functional biomaterials including stimuli-responsive hydrogels, antioxidant nanocarriers, and controlled-release systems, one may enhance their stability and sustained bioactivity in pathological tissues. Such strategies may be particularly beneficial in addressing lesions with high microenvironmental sensitivity, including chronic wounds and fibrotic kidney tissues.

Furthermore, to address the heterogeneity among diabetic patients, developing predictive biomarkers and individualized strategies is essential. Screening systems based on metabolic profiles and treatment responses, coupled with omics-integrated machine learning models, can further improve patient stratification and optimize clinical trial design.

Finally, to support clinical translation, more pathophysiologically relevant animal models that recapitulate the chronic nature of diabetes and its complications should be employed. Long-term, multi-dose exposure studies are needed to comprehensively evaluate safety, immunogenicity, and metabolic effects. Simultaneously, the establishment of GMP-compliant production pipelines and standardized quality control procedures is critical for enabling the scalable production and regulatory approval of PENP-based therapies.

In summary, future investigations of PENPs in the treatment of diabetes and its complications should adopt a fully integrated and systematic approach encompassing platform optimization, mechanistic elucidation, targeted delivery, multi-targeted combination therapy, and clinical validation. Through interdisciplinary collaboration and close integration of preclinical and clinical studies, a precision therapeutic paradigm centered on PENPs can be established, offering a practical and effective solution for the comprehensive management of diabetes and its complications.

Practically, we propose a staged roadmap comprising three phases. In the first phase, dose–response relationships, biodistribution profiles, and pharmacokinetic baselines of PENPs should be established across at least two diabetic models under harmonized endpoints. In the second phase, repeat-dose toxicology and the immunogenicity of PENPs are expected to be evaluated with prespecified stopping rules and predefined potency criteria. In the third phase, GMP-compliant manufacturing processes for PENPs need to be finalized and validated with defined release specifications, and reporting aligned with MISEV and EV-TRACK ought to be implemented to facilitate regulatory engagement.

## 11. Conclusions

Current evidence indicates that PENPs hold considerable potential as bioactive nanomaterials for diabetes therapy. Although a wide range of studies have demonstrated beneficial effects on oxidative stress, inflammation, metabolic regulation, and tissue repair, the majority of available data remain confined to preclinical models. Moreover, heterogeneity in plant sources, dosing units, and experimental protocols continues to limit the reproducibility and comparability of findings. Future progress will depend on methodological standardization across isolation, purification, and characterization procedures, as well as on the establishment of rigorous quality control systems capable of addressing batch-to-batch and source-related variability. Particular attention should be devoted to systematic investigations of dose–response relationships, pharmacokinetic and biodistribution profiles, and long-term safety under repeat administration since these aspects remain insufficiently documented. To accelerate translation, research efforts need to achieve defined milestones that include reproducible pharmacological outcomes in multiple diabetic models under harmonized endpoints, validated criteria for repeat-dose toxicology and immunogenicity, and the development of GMP-compliant manufacturing processes with clearly defined release specifications. Alignment with international reporting standards such as MISEV and EV-TRACK will further enhance transparency and regulatory preparedness. By addressing these scientific and translational challenges, PENPs may evolve from experimental observations into clinically testable nanotherapeutics with the potential to advance the precision management of diabetes and its complications.

## Figures and Tables

**Table 1:** Comparative lipid profiles of PENPs from different plant sources and their correlation with potential in vivo targeting.

Plant Sources	Dominant Lipid Species and Composition	Potential In Vivo Targeting	Ref.
Grapefruit	PE (~46%), PC (~29%)	Intestine (enhanced uptake), liver (preferential accumulation)	[64]
Ginger	PA (~38%), DGDG (~33%), MGDG (~21%)	Small intestine (uptake by epithelial cells, oral)	[65]
Oat	PC (~30%), DGDG (~29.8%)	Brain (crosses BBB; microglial uptake)	[66]

**Table 2:** Comparative trade-offs of PENP extraction and purification methods in terms of purity, scalability, and vesicle integrity.

Method	Isolation Principle	Purity	Scalability	Vesicle Integrity	Advantages	Limitations
Differential centrifugation (DC)	Sequential centrifugation with gradually increasing g force to pellet particles	Low to moderate	High	Moderate to low	Simple, low cost, widely accessible	High risk of co-pelleting proteins and organelle fragments, variability between batches
Density-gradient ultracentrifugation (DGUC)	Separation by buoyant density in sucrose or iodixanol medium	High	Low	Moderate	High resolution and purity	Time-consuming, low-throughput, possible osmotic stress
Ultrafiltration (UF, including TFF)	Filtration based on membrane pore size and continuous flow	Moderate	High	Moderate to high	Suitable for large volumes, relatively fast	Membrane fouling, loss of small vesicles
Size-exclusion chromatography (SEC)	Gel filtration to separate vesicles from proteins and small molecules	High	Moderate	High	Gentle on vesicles, preserves bioactivity	Limited-throughput, dilution and reduced yield
Polyethylene glycol precipitation (PEG)	Polymer induced precipitation of vesicles	Low to moderate	High	Moderate to low	Rapid, inexpensive, scalable	Co-precipitation of contaminants, polymer residues
Immunoaffinity capture	Isolation based on specific vesicle surface markers such as TET8 or PEN1	Very high	Low	High	High specificity for target subpopulations	High cost, dependence on antibody availability, limited yield
Asymmetric flow field-flow fractionation (AF4)	Fractionation of vesicles by size under a flow field	High	Low to moderate	High	High resolution, maintains structural integrity	Specialized instrumentation, method complexity
Aqueous two-phase system (ATPS)	Partitioning of vesicles between two immiscible polymer phases	Moderate to high	High	High	Gentle on vesicles, potentially scalable	Need for removal of residual polymers, optimization required
Electrophoresis–dialysis hybrid	Use of electric field with dialysis membrane to remove charged impurities	High	Low	High	Effective for charged impurities, precise separation	Low-throughput, labor-intensive
Microfluidic platforms	On-chip vesicle sorting by hydrodynamic or affinity based principles	High	Low to moderate	High	Rapid, automated, requires minimal sample	Scale up not established, device-specific variability

**Table 3:** Comparative summary of PENP biodistribution and disease applicability under different administration routes.

Route of Administration	Plant Sources	Primary Target Organs/Tissues	Key Advantages	Applicable Disease Models	Ref.
Oral Administration	Ginger, Grapefruit, Ginseng	Distal small intestine, cecum, colon, liver	High stability, strong dependence on the enterohepatic axis	Inflammatory bowel disease, intestinal cancer, non-alcoholic fatty liver disease	[98,109,110]
Intravenous Administration	Ginseng, Corn	Liver, spleen, systemic circulation	Bypasses first-pass metabolism, high systemic delivery efficiency	Cancer, liver diseases, cerebral ischemia, and other systemic diseases	[111,112]
Intraperitoneal Administration	Grapefruit, Ginseng	Liver, spleen, kidneys, lungs	Stable pharmacokinetics, suitable for animal studies	Inflammation, systemic immune-related diseases	[47,70,111]
Intranasal Delivery	Grapefruit	Lungs, brain	Non-invasive, bypasses the BBB	Brain tumors, neuroinflammation, pulmonary diseases	[70,114]
Transdermal Delivery	Safflower, Ginseng	Dermis, subcutaneous tissue	Targeted to wound sites, suitable for localized therapy	Wound healing, chronic dermatitis	[113]
